# Microbial Derived Compounds, a Step Toward Enhancing Microbial Inoculants Technology for Sustainable Agriculture

**DOI:** 10.3389/fmicb.2021.634807

**Published:** 2021-02-18

**Authors:** Judith Naamala, Donald L. Smith

**Affiliations:** Smith Laboratory, Department of Plant Science, McGill University, Quebec, QC, Canada

**Keywords:** plant growth promoting microorganisms, microbe derived compounds, sustainable agriculture, phytomicrobiome, stress

## Abstract

Sustainable agriculture remains a focus for many researchers, in an effort to minimize environmental degradation and climate change. The use of plant growth promoting microorganisms (PGPM) is a hopeful approach for enhancing plant growth and yield. However, the technology faces a number of challenges, especially inconsistencies in the field. The discovery, that microbial derived compounds can independently enhance plant growth, could be a step toward minimizing shortfalls related to PGPM technology. This has led many researchers to engage in research activities involving such compounds. So far, the findings are promising as compounds have been reported to enhance plant growth under stressed and non-stressed conditions in a wide range of plant species. This review compiles current knowledge on microbial derived compounds, taking a reader through a summarized protocol of their isolation and identification, their relevance in present agricultural trends, current use and limitations, with a view to giving the reader a picture of where the technology has come from, and an insight into where it could head, with some suggestions regarding the probable best ways forward.

## Introduction

The holobiont of terrestrial plants, defined as the plant and its associated phytomicrobiome (Hartmann et al., 2014), is estimated to be nearly half a billion years old (Knack et al., 2015). The coexistence of both plants and microbes is largely dependent on a cascade of chemicals produced by both partners, as a means of communication (signals), source of food or simply as survival mechanisms, e.g., competition (antibiotics, antifungals, etc.). The plant almost always regulates the composition of the phytomicrobiome, especially in its rhizosphere, depending on its condition and that of its surroundings, mostly, through the type of exudates it produces. Among the phytomicrobiome are organisms that can promote plant growth, which are commonly referred to as, plant growth promoting microorganisms (PGPM) (Bender et al., 2016; Naamala and Smith, 2020). PGPM are very diverse, (Naamala et al., 2016; Fan et al., 2018), with substantial numbers of strains, from varying species and genera, largely bacteria and fungi. A number of PGPM have been isolated from the rhizosphere and plants, for use in inoculant production, for enhanced crop production (Bashan et al., 2014). Use of PGPM, as inoculants, in crop production is a common and old practice (Bashan et al., 2014) in many parts of the world, for increased productivity and sustainability (Babalola and Glick, 2012). There is currently a substantial number of promising microbial inoculants, some already on the market (Velivelli et al., 2014; Mehnaz, 2016; Berninger et al., 2018; Arthur and Dara, 2019), with various mechanisms of enhancing crop growth, ranging from growth stimulation, to enhanced defense against pathogens and abiotic stress (Barea, 2015; Gupta et al., 2015; Bender et al., 2016; Msimbira and Smith, 2020; Naamala and Smith, 2020). Despite their undisputable success in enhancing crop production (de Boer et al., 2019; Lyu et al., 2020), the use of PGPM technology, in crop production, has been constrained by a number of limitations, most notably, inconsistencies, especially under field conditions. The latter notwithstanding, contributions of PGPM to organic crop production and soil productivity remain valuable approaches, especially, given ongoing climate change, and the resulting rendering of many agricultural soils as unfit for crop production. There is also a drive to minimize extensive use of chemicals in agricultural production, due to effects on the environment and human health, related to their use. Therefore, there is need to address limitations related to PGPM technology, allowing for more successful use. The most recent approach is the use of PGPM derived compounds as alternatives, supplements, or complements to microbial cells. It has caught the attention of many researchers and industrial partners, who believe that compounds could, in one way or the other, address some of the limitations associated with use of PGPM inoculants. Despite studies on microbe derived compounds being somewhat slow (Lemfack et al., 2014), in part due to the complexity in structure and properties of some compounds, such as volatile organic compounds, there are a number of promising research findings showing the ability of some microbe derived compounds to positively impact plant growth, and mitigate abiotic and biotic stress, that would otherwise affect plant growth and productivity (Miransari et al., 2013).

## Microbial Derived Compounds

### Brief Background

Despite modern technology and equipment, a lot is yet to be uncovered about the phytomicrobiome of both domesticated and undomesticated plants (Lyu et al., 2020), partly due to their inability to grow/be cultured outside their natural environment. As a result, a lot is yet to be learned regarding microbial derived compounds. Microbial derived compounds are mostly secondary metabolites (Gray et al., 2006; Schulz and Dickschat, 2007; Schmidt et al., 2015; Piechulla et al., 2017; Schulz-Bohm et al., 2017), that are excreted by microorganisms, in response to known and unknown stimuli, such as, nutrient deficiency, competition for niche space, or even, signals from a host plant, etc. Such secondary metabolites may include; hormones, volatile organic compounds (VOCs), enzymes, antimicrobials, siderophores, etc. (Crowley et al., 1988; Bais et al., 2006; Dimkpa et al., 2009; Lemfack et al., 2014, 2018), which may serve a range of functions for the producer (microbe) and receiver (another microbe or plant). For instance, lipochitooligosaccharides (LCO) are symbiotic signals in both rhizobia (nod factors) (Schultze and Kondorosi, 1996; Miransari et al., 2013) and arbuscular mycorrhizae (Myc factors), from the microbes to their host plants (Maillet et al., 2011; Tanaka et al., 2015). The role they play in the legume rhizobia symbiosis is reasonably well studied. During the legume-rhizobia symbiosis, once perceived by the host legume plant, LCO triggers curling of the root hairs among other physiological changes that occur in the root of the host plant (Souleimanov et al., 2002; Prithiviraj et al., 2003; Miransari et al., 2013), a process that initiates nodule formation (Schultze and Kondorosi, 1996; Miransari et al., 2006). Until recently, the ability of LCO to directly enhance plant growth was unknown. Gram negative bacteria produce N-Acyl-L-Homoserine Lactones (AHLs) to monitor and manage their populations through quorum sensing (Ortíz-Castro et al., 2009; Liu et al., 2012, 2020; Schenk and Schikora, 2014; Hanifa et al., 2020; Shrestha et al., 2020). Some compounds, such as siderophores and bacteriocins, are produced to give the producer an upper hand during competition for resources such as nutrients and niche space, respectively. Fungal-produced VOCs are believed to play a part in mycelia growth and sporulation (Ortíz-Castro et al., 2009). On the other hand, thuricin17 is a relatively new compound, whose production by *Bacillus thuringiensis* NEB17, is encoded by 3 copies of the same gene (Nazari and Smith, 2020). It is a class IId bacteriocin, which inhibits growth of some bacteria, that could otherwise compete with it for resources (Nazari and Smith, 2020). Whatever the reason for producing a particular compound may be, to the microbe, research has shown that plants have evolved mechanisms of perceiving some of these compounds (Veliz-vallejos et al., 2014), and that they can actually enhance plant growth (Banchio et al., 2009), under stressed and non-stressed conditions (Dyachok et al., 2002; Duzan et al., 2005; Barriuso et al., 2008; Miransari and Smith, 2009; Schenk et al., 2012; Hanifa et al., 2020; Shrestha et al., 2020).

### Role of Microbe Derived Compounds in Plant Growth

Microbe derived compounds play a range of roles in plant growth, ranging from direct enhancement of plant growth, to mitigation of biotic and abiotic stress, plus bioremediation. Below are some of the roles that have been reported for some compounds with regard to plant growth enhancement.

#### Stimulation of Plant Growth

Microbe derived compounds can stimulate plant growth directly, through increasing plant biomass, root length (Souleimanov et al., 2002), germination rate (Subramanian et al., 2016), etc., or even increasing availability and uptake of nutrients by the plant (Crowley et al., 1988). For instance recent studies have shown that LCOs can enhance growth of many crop species, under stressed and non-stressed conditions (Souleimanov et al., 2002; Atti et al., 2005; Duzan et al., 2005; Miransari et al., 2006; Khan et al., 2011; Kidaj et al., 2012; Prudent et al., 2015; Schwinghamer et al., 2015; Subramanian et al., 2016; Arunachalam et al., 2018). Prudent et al. (2016) reported that it enhances nodule formation and N supply. LCO also increased plant biomass in *Glycine max* and *Zea maize* and root length in *G. max* (Souleimanov et al., 2002). On the other hand, thuricin17 is a new compound, whose production by *Bacillus thuringiensis* NEB17, is encoded by 3 copies of the same gene (Nazari and Smith, 2020). It doubles as both a class IId bacteriocin (which inhabits growth of some bacteria) and plant growth stimulant (Nazari and Smith, 2020). Thuricin17 has also been reported to enhance growth of a range of crop species under stressed and non-stressed conditions (Lee et al., 2009; Prudent et al., 2015; Subramanian et al., 2016). For instance, plant growth stimulation in *Zea mays* and *Panicum virgatum* (Lee et al., 2009; Arunachalam et al., 2018) were observed. Subramanian et al. (2016) observed an increase in germination of soybean seeds inoculated with LCO and thuricin17. and induction of defense related enzymes in *Glycine max* (Jung et al., 2011), were observed. The compound (thuricin17) has no effect on useful nitrogen fixing rhizobia and other plant growth promoting bacteria (Gray, 2005). This, coupled with the compound’s high tolerance to denaturation on relatively low temperatures and a wide range of pH, make thuricin17 a hopeful candidate for use in sustainable agriculture. Some compounds, notably VOCs, have been reported to enhance plant quality through enhanced accumulation of aromatic compounds (Banchio et al., 2009). An increased accumulation and emission of R-terpineol and eugenol essential oils was observed *Ocimum basilicum* (sweet basil) plants treated with a *Bacillus subtilis* strain that released VOCs (Banchio et al., 2009). VOCs have also been reported to enhance plant growth, through making nutrients such as sulfur more available (Meldau et al., 2013). Treatment of *Nicotiana attenuata* plants with a volatile compound, Dimethyl disulfide (DMDS) emitted by *Bacillus* sp. strain B55, eliminated plant growth limitation as a result of inadequate sulfur (Meldau et al., 2013). In addition to enhancing plant growth, compounds can also enhance growth of beneficial phytomicrobiome in the soil.

#### Mitigation of Biotic Stress Related Effects

Biotic stress includes living things like weeds, insect pests, and pathogens that negatively impact plant growth. While chemicals have been and are still being widely used to control biotic stress, in many parts of the world, minimizing their use is being encouraged, due to negative effects such as soil and water contamination, that are related to their use. Biocontrol has proven to be a promising approach to managing biotic stress in agriculture. Compounds mitigate biotic stress in various ways. For instance, some VOCs were reported to enhance growth of useful microbial population in the rhizosphere, or enhance important characteristics such as biocontrol, in some bacteria (D’Alessandro et al., 2014; Schulz-Bohm et al., 2017). There was an increase in the number of *Cotesia marginiventris* (a parasitoid that attacks a maize pest *Spodoptera littoralis*) in soils treated with 2,3-butanediol, a compound produced by *Enterobacter aerogenes* (D’Alessandro et al., 2014), although application of the compound had no direct effect on the pest. Some compounds can directly suppress plant pathogens (Kai et al., 2009; De Vrieze et al., 2015), induce systemic resistance (Song and Ryu, 2013; Choi et al., 2014; D’Alessandro et al., 2014; Wintermans et al., 2016) and/or induce soil fungistasis and suppressiveness (van Agtmaal et al., 2015). For instance, maize plants treated with 2,3-butanediol, were more resistant against the fungus *Setosphaeria turcica*, a causative agent of Northern corn leaf blight (D’Alessandro et al., 2014). 3-pentanol, reduced severity of *Xanthomonas axonopodis* and cucumber mosaic virus, in *Capsicum annuum* L. cv. Bukwang, under field conditions (Choi et al., 2014). Song and Ryu (2013), observed an increase in *Coccinella septempunctata* lady beetle, a natural enemy of *Myzus persicae*, in *Cucumis sativus* L. cv. backdadagi) treated with VOCs 3-pentanol and 2-butanone, leading to a decrease in the aphids’ population. The same authors observed that the two VOCs induced ISR against *Pseudomonas syringae pv. Lachrymans* and an increase in the fresh weight of cucumber fruits, under field conditions (Song and Ryu, 2013). Other compounds may enhance nutrient availability for plant uptake (Crowley et al., 1988; Meldau et al., 2013) or induce plant production of secondary metabolites beneficial to the plant (Santoro et al., 2011), hence increasing the plant’s ability to thrive amidst biotic stress challenges. Prudent et al. (2016) reported that LCO enhances nodule formation and N supply. Mitigating biotic stress could be a result of the compound directly suppressing pathogens and or through induced systemic resistance, as well as improving soil characteristics such as fungistasis (Ryu et al., 2003; Bais et al., 2006; Zhang et al., 2008; El-Hasan and Buchenauer, 2009; Jung et al., 2011; Huang et al., 2012; De Vrieze et al., 2015; Tahir et al., 2017; de Boer et al., 2019). Duzan et al. (2005) reported that LCO enhances resistance to *Microsphaera diffusa* in *Glycine max*. Nematicidal volatiles such as 2-undecanone and dimethyl disulphide, produced by *Bacillus megaterium* YMF3.25 lowered the egg hatching rate, and infection of *Meloidogyne incognita*, in a petri plate experiment (Huang et al., 2010). VOCs, such as 2,3-butanediol, produced by three strains of *Bacillus subtilis* inhibited growth of *Fusarium oxysporum* f.sp. *radices lycopersici* mycelia (Baysal et al., 2013).

#### Mitigating Abiotic Stress Related Effects

Abiotic stress such as salinity, drought, floods and acidity are a major constraint in agricultural production. Large areas of arable land have been rendered unproductive (Naamala et al., 2016). Microbial derived compounds play a vital role in elimination of abiotic stress effects on plants. For instance, Long-chained AHL compounds produced by *Burkholderia graminis*, were reported to enhance both growth and salt tolerance in tomato (Barriuso et al., 2008). Siderophores produced by *Streptomyces acidiscabies* E13 alleviated metal induced oxidative stress in cowpea plants (Dimkpa et al., 2009). Zhao et al. (2020) reported enhanced tolerance to salt by *Arabidopsis thaliana* plants treated with N-3-oxo-hexanoyl-homoserine lactone. Regulation of biomass and leaf arrangement in drought stressed *Brassica napus* [L.], treated with LCO was observed (Schwinghamer et al., 2016). Subramanian et al. (2016) observed an increase in germination percentage of soybean seeds exposed to salinity, somewhat similar to non-stressed seeds, following treatment with LCO and thuricin17. Atti et al. (2005) reported that it enhances *Glycine max* growth under water stress conditions. *Brassica napus* [L.] germination was increased by 75% following addition of LCO, under low temperature stress (Schwinghamer et al., 2015).

#### Bioremediation of Xenobiotic Compounds

Xenobiotic compounds such as organophosphates, aromatic hydrocarbons, phenols and heavy metals, are a major source of soil and environmental degradation in many parts of the world (Cameotra and Bollag, 2003; Jha et al., 2015; Gangola et al., 2019; Thakur et al., 2019). They are considered potentially toxic, carcinogenic and persist in the soil for long periods of time. They are introduced in agricultural soils, largely through use of chemicals such as pesticides, fertilizers and herbicides (Jha et al., 2015; Gangola et al., 2019). Industrialization, especially pharmaceutical companies and mining also play a major role in introducing xenobiotics to the environment. Given the growing industrialization and the current heavy use of chemicals, especially in agriculture (Gangola et al., 2019), it is important that a viable and sustainable approach to degrade such compounds is developed (Jha et al., 2015). Use of physical and chemical approaches has proven to be costly (Gangola et al., 2019). Use of biological approaches has been considered a potential relatively cheaper and sustainable approach (Gangola et al., 2019). Microbial species with bioremediation properties have been reported by researchers (Cameotra and Bollag, 2003; Gangola et al., 2019). Many of such species produce compounds, such as enzymes and biosurfactants, as a bioremediation mechanism. Enzymes, such as organophosphate hydrolase (OpdA), that was isolated from *Agrobacterium radiobacter* (Horne et al., 2002) and SsoPox, isolated from *Sulfolobus solfataricus*, were reported as able to degrade xenobiotic compounds such as organophosphates (Cameotra and Bollag, 2003; Hiblot et al., 2012; Thakur et al., 2019). a number of organophosphate pesticides, through hydrolyzation (Thakur et al., 2019). The ability of *Pseudomonas* sp. to degrade ADP has been associated with its possession of the enzyme atrazine chlorohydrolase (Jha et al., 2015). Slowness and effect of environmental conditions on the microbe have been reported as potential limitations to use of microbial cells in bioremediation (Gangola et al., 2019). Direct use of compounds may address such limitations.

**FIGURE 1:**
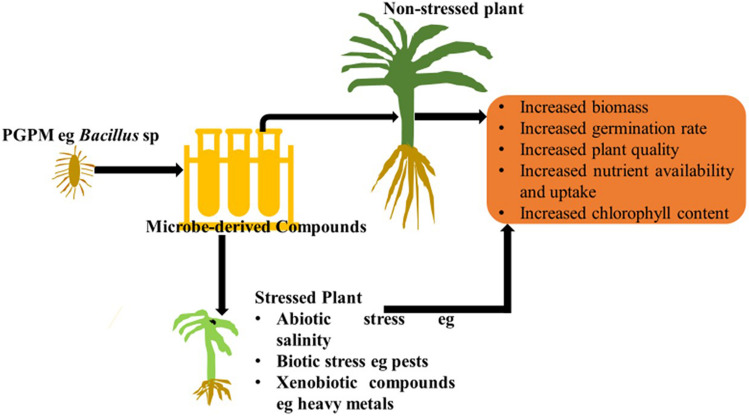
The role microbial derived compounds play in enhancing plant growth and plant quality.

Figure 1 and Table 1 below summarize the role compounds play in enhancing plant growth.

**TABLE 1:** Showing the role compounds play in enhancing plant growth.

Function	Compounds involved	References
Mitigation of pathogens	Acetoin, 2-Phenyl-ethanol, 2-butanone, 2-Non-anone, B -Caryophyllene, 2S,3S butandiol, Benzothiazole, C4-HL, C6-HL, Tensin, Viscosinamide, 3-oxo-C14- HSL; 3OC6-HSL, 3-oxo-C16:1-HL	Nielsen et al., 1999; Thrane et al., 2000; Mathesius et al., 2003; Ryu et al., 2003; Schuhegger et al., 2006; Zhang et al., 2007; Arrebola et al., 2010; Groenhagen et al., 2013; Song and Ryu, 2013; D’Alessandro et al., 2014; Schenk et al., 2014; De Vrieze et al., 2015; Raza et al., 2016; Shrestha et al., 2019; Liu et al., 2020
Plant growth stimulation	1-Undecene, Acetophenone, 2-Methyl-n-1-tridecene, 1-Butanamine, Benzaldehyde, 4-Nitroguaiacol, m. Cymene, C10-HL, C6-HL, C8-HL, 3-OH-C10-HL, Thuricin17, LCO,	De Jong et al., 1993; Souleimanov et al., 2002; Delalande et al., 2005; Almaraz et al., 2007; Khan et al., 2008, 2011; Lee et al., 2009; Gutiérrez-Luna et al., 2010; Minerdi et al., 2011; Velazquez-Bererra et al., 2011; Bai et al., 2012; Kidaj et al., 2012; Veliz-vallejos et al., 2014; Tanaka et al., 2015; Fincheira et al., 2016; Han et al., 2016; Prudent et al., 2016; Rankl et al., 2016; Schwinghamer et al., 2016; Vaishnav et al., 2016; Schulz-Bohm et al., 2017
Enhance plant nutrient availability and acquisition	Dimethyl disulfide, Ferrioxamine B (FOB); LCO	Crowley et al., 1988; Prithiviraj et al., 2003; Meldau et al., 2013; Prudent et al., 2016
Mitigation of weeds	Anisomycin, Herboxidiene, Phosphinothricin	Duke and Lydon, 1987; Isaac et al., 1992; Saxena and Pandey, 2001
Suppression of pests	Cyclo(L-Leu-L-Pro), Avermectin	Tanaka and Omura, 1993; Song et al., 2017
Mitigation of abiotic stress	C14-HL, Thuricin17, LCO; 3OC6-HSL	Atti et al., 2005; Miransari et al., 2006; Barriuso et al., 2008; Prudent et al., 2015; Subramanian et al., 2016; Zhao et al., 2020

## Isolation, Purification, and Identification of Microbial Compounds

In the soil, the composition and quantity of microbial compounds produced is dependent on abiotic and biotic factors, notably, moisture, temperature, pH, soil texture, and the soil microbial community (Schmidt et al., 2015; Potard et al., 2017; Raza et al., 2017; van Agtmaal et al., 2018; de Boer et al., 2019; Kramshøj et al., 2019). Because the soil environment cannot be easily controlled, the same is true for what and when a given compound can be produced by a given microbe. However, under laboratory conditions, it is possible to have significant control over what, how much and when a compound can be produced, by controlling the microbe’s growth environment and composition. The composition of microbial compounds produced in artificial cultures can be influenced by whether the microbial culture is pure or a consortium (Schulz-Bohm et al., 2015; Tyc et al., 2015, 2017; Kai et al., 2018). Even though it is a long process, and complicated for some compounds, new technology has made it possible to obtain and identify compounds of culturable microorganisms, in laboratories. Invention and advances in techniques such as tandem mass spectrometry and nuclear magnetic resonance (NMR) spectroscopy have eased analysis and identification of obtained microbial compounds (Armengaud, 2013; Kucharova and Wiker, 2014; Otto et al., 2014). Chromatography, such as high pressure liquid chromatography is also at the forefront of separation and purification of compounds (Gray et al., 2006). There is no single universal protocol for obtaining microbial compounds. Although most steps can be similar, there may be variations right from culturing of microbes, to the type and concentration of chemicals used in compound isolation, by different laboratories. In our laboratory, we follow procedures by Gray et al. (2006) with a few modifications, depending on the microbe being dealt with. Below, are general steps of the procedure. The microbe of interest is cultured in appropriate media, under appropriate conditions, for a given period, depending on the growth type of the microbe. For instance, 48 h for fast growing bacteria. After incubation, centrifugation is conducted at 10,000 rpm and 4°C, for 10 min (Gray et al., 2006). This step aides the separation of microbial cells from the cell-free supernatant. Filtration then follows, using an appropriate filter, to eliminate any chance of contaminating the supernatant with microbial cells. Usually, a filter with a size pore of 0.22 μm is appropriate for bacteria, and some fungi. As mentioned above, a number of factors influence production of compounds. It is therefore important to carry out a bioassay, using the obtained cell-free supernatant, to be sure of the presence of bioactivity. It is vital that appropriate controls are used during the experiment, to eliminate the possibility of anything else but a microbial compound as source of bioactivity. Once bioactivity is confirmed, the supernatant is subjected to appropriate chromatogragy, such as high pressure liquid chromatography (HPLC), for liquids, or gas chromatography for gasses, to obtain peaks. Peaks are then collected, purified, and tested for biological activity. A peak with a positive bioassay is then subjected to fractionation using appropriate carrier compounds such as acetonitrile, and water. Each fraction is assessed for bioactivity. The fraction with a positive bioassay is then used for appropriate chromatography evaluation, such as HPLC on a Vydac C18 reversed-phase column (0.46 × 25 cm, 5 μm) at 214 nm with gradient from 5 to 95% acetonitrile. The HPLC fractions are then collected, freeze dried and, again, tested for biological activity. The biologically active HPLC fraction containing one chromatographic peak is then used for identification of the active compound, using appropriate techniques, such as mass spectrometry (Barriuso et al., 2008). It should be reiterated that there are many protocols one can follow from culturing the microbe, to identification of a compound. A reasonable number of compounds have already been discovered. For instance, there are approximately 2000 microbial VOCs, from approximately 600 microorganisms, especially bacteria and fungi, with varying chemical and molecular structures and forms, such as, fatty acid derivatives, alcohols, and ketones (Schulz and Dickschat, 2007; Lemfack et al., 2014, 2018; Schenkel et al., 2015; Schmidt et al., 2015; Schulz-Bohm et al., 2017; de Boer et al., 2019).

### Mode of Application of Microbe Derived Compounds, on Plants

There are various ways through which compounds can be applied to the host plant. One of them, is through spraying the aerial part of the plant, such as leaves and stems (Atti et al., 2005; Arunachalam et al., 2018). The compound may also be drenched in the soil, near the plant roots, or seedlings and seed soaked in the compound treatment (Duzan et al., 2005; Bai et al., 2012; Song and Ryu, 2013; Choi et al., 2014).

## Modes of Action

Although more needs to be understood, especially about how some plants perceive microbial compounds, different modes of action, through which compounds enhance plant growth have been suggested by some researchers (Bai et al., 2012; Piechulla et al., 2017). The mode of action employed may differ from one compound to another, with some compounds possessing more than one mode of action.

Some compounds function by activation of genes responsible for production of certain phytohormones such as auxins and cytokinins (Zhang et al., 2007; Contreras-Cornejo et al., 2009; Bai et al., 2012; Prudent et al., 2016; Piechulla et al., 2017), activation of enzymes and genes involved in disease resistance (Duzan et al., 2005; Choi et al., 2014) and enhancing production of enzymes and genes, essential in stress management (Jung et al., 2011; Zhou et al., 2016; Zhao et al., 2020) through processes such as inhibition of reactive oxygen species (ROS) production by plant cells (Blom et al., 2011). For example, a study by Jung et al. (2011) indicated that thuricin17 induced defense related enzymes in soybean leaves. Bai et al. (2012) showed that N-3-oxo-decanoyl-homoserine-lactone (3-O-C10-HL) stimulated the expression of auxin-response genes in seedlings of *Vigna radiata*, resulting in formation of auxin-dependent adventitious roots. AHLs have also been reported to enhance upregulation of defense and stress management proteins (Mathesius et al., 2003), metabolites such as proline (Zhao et al., 2020), and genes such as *COR15a*, *RD22*, *ADH*, and *P5CS1* (Zhao et al., 2016, 2020) as well increased activity of defense related enzymes such as peroxidases and catalases (Piechulla et al., 2017; Liu et al., 2020). A study by Choi et al. (2014) showed that field pepper (*Capsicum annum*) plants treated with 3-pentanol, a VOC produced by *Bacillus amyloliquefaciens* strain IN937a, showed an increased expression of proteins CaPR1 and CaPR2, involved in *capsicum annum* pathogenesis, as well as Ca protease inhibitor2 (CaPIN2). Some compounds, once taken up by plants, undergo processes which result in plant growth stimulation. For instance, AHL amidolysis by amide hydrolase (FAAH), a plant-derived fatty acid, to yield L-homoserine, which in low concentrations, stimulates plant growth (Palmer et al., 2014). Some compounds may simply result in increased accumulation of phenolic compounds, such as salicylic acid, in plants (Schenk et al., 2014).

The mode of action may be influenced by factors such as plant and microbe genotype, environmental factors such as soil moisture and temperature, as well as the type and concentration of the compound (D’Alessandro et al., 2014; Palmer et al., 2014; Shrestha et al., 2019, 2020). For instance, while long chained AHLs induced tolerance to *Pseudomonas syringae* pv. in tomato, short chained AHLs had no effect on the same crop, even in combination with long chained AHLs (Schenk et al., 2014; Shrestha et al., 2020). However, Liu et al. (2012), reported that, short chained (C6 and C8) AHLs enhanced elongation of the primary root in *Arabidopsis thaliana*, while long chains (C12 and C14) inhibited root elongation. Almaraz et al. (2007), showed that out of four LCOs used in their study, only NodBj-V (C18:1, MeFuc) had significant effects on soybean growth. A single compound may possess more than one mode of enhancing growth of a single or multiple plant species (Schulz-Bohm et al., 2017) and sometimes, a compound which enhances growth of one plant species may inhibit growth of another, similar to one which suppresses a pathogen may also suppress growth of beneficial microorganisms.

## Relevance of Microbial Compounds

The rhizosphere is an environment with a diversity of microorganisms as a resource, which, if properly tapped, could enhance the already promising PGPM technology. Tapping this should not be limited to microbial cells, but also their by-products, such as compounds, which are already showing promising results. Acquisition of microbial compounds is a longer process compared to direct use of microbial cells, which would leave one wondering if they should simply settle for the latter. However, there are circumstances under which direct use of compounds would be relevant and perhaps more beneficial than microbial cells.

### Reliability and Easy to Control Quantity and Quality of a Compound of Interest

For instance, even though compounds are produced by microbes, there are several factors which influence the type and concentration of a compound produced by a microbe (Schmidt et al., 2015). Given the dynamic state of the soil environment, with soil conditions frequently changing, it can never be guaranteed that, for a particular added microbe, a specific compound of interest will be produced. It should also be noted that under field conditions, there are inter- and intra-species interactions which may also influence the type and concentration of compounds produced by a microbe (van Agtmaal et al., 2018). The target stress may also play a role in the effectiveness of the compound, given that, for instance, some soil pathogens are more sensitive to certain compounds than others (van Agtmaal et al., 2018). This could be one of the causes of inconsistencies observed in the field, following the use of PGPM technology. It is relatively easier to control the growth environment of a microbe under artificial conditions, which makes for a more reliable and certain way of obtaining a compound of interest, moreover, in larger quantities that could be utilized even in areas where the microbe may not establish and colonize, or at least not to a sufficient degree. There are also reports of compounds stimulating plant growth at one stage but not the other (El-Hasan and Buchenauer, 2009). For instance, germination of maize seeds, on filter paper enriched with 200 and 300 mg L^–1^ 6-pentyl-alpha-pyrone was negatively affected, while the same amount of the compound applied on seedlings of the same plant, enhanced seedling growth (El-Hasan and Buchenauer, 2009). Directly applying a compound could eliminate the possibility of an appropriate compound being produced at the wrong time, or even the right time but in wrong concentrations (either too high or too low). It should be noted that most of these compounds are required in very low concentrations for beneficial effects. High concentrations tend to antagonize plant growth (Lo Cantore et al., 2015).

### Minimize Risk of Pathogenicity

Some PGPMs such as *Pseudomonas aeruginosa* are opportunistic pathogens causing disease in some plant species. Isolating compounds and applying them directly may minimize the risk associated with their pathogenicity in the field. This would make processes such as crop rotation and mixed cropping less risky in terms of disease spread. Further, some microbes, such as, *Pseudomonas simiae* produce both growth promoting and growth inhibiting compounds. Directly applying the growth promoting compound could lower risks of exposing plants to inhibiting compounds, which might be likely, if the microbe instead of compound was applied. For instance, *Pseudomonas simiae* produces volatile organic compounds quinoline and 1-undecene. At the same concentration, 1-undecene enhanced germination of soybean seeds, while quinoline inhibited germination of the same seeds (Vaishnav et al., 2016).

### Possible Benefits of a Broader Range of Hosts

Some PGPM are host specific while the compounds they produce, if applied directly, can benefit a wider range of crop species. For instance, *Bradyrhizobium japonicum* produces LCOs that are essential in initiation of nodulation in host legume plants. In this context, LCO will only benefit the host plant. Assuming that, once released by the *Bradyrhizobium japonicum*, LCO confers benefits to neighboring plants of different species, this still has limited space in the current trend of expanding monoculture. Even in mixed crop systems, associated crops may not benefit, for as long the host plant is not a crop species. Fortunately, isolation of LCO makes it possible to benefit other crops, in diverse ways, even in the absence of soybean, or *Bradyrhizobium japonicum* (Atti et al., 2005; Duzan et al., 2005; Miransari et al., 2006; Khan et al., 2011; Kidaj et al., 2012; Subramanian et al., 2016).

### Increased Effectiveness

Because they are living organisms, microbes tend to be affected by conditions such as drought, salinity (Miransari et al., 2013), antibiotics (Naamala et al., 2016), and aluminum toxicity (Jaiswal et al., 2018), among others (van Agtmaal et al., 2018), to the extent that they can be rendered ineffective (van Agtmaal et al., 2015) in promoting plant growth. For instance, during their experiment, van Agtmaal et al. (2015) observed an absence of pathogen suppressive VOCs in soil assays with exposure to anaerobic disinfestation stress, as compared to unstressed soils (van Agtmaal et al., 2015). The VOCs were observed again after 15 months. In such cases, compounds isolated under optimum conditions can be applied to enhance plant growth under stressed conditions, after all, some compounds have been found effective only when a plant has been exposed to stressful conditions. Therefore, where microbes may not be effective, compounds could be applied to enhance plant growth, or mitigate effects of abiotic stress on vital processes such as the legume-rhizobia symbiosis.

### Less Costly and Easier to Handle

Also, compounds required in very small quantities, are less costly and easy to store than microbial cells, they can be making the former more affordable and easier to handle. Given these factors microbe derived compounds are clearly relevant in today’s and potentially future agricultural practices. However, under circumstances where both PGPM cells or their derived compounds can be used, the question of whether to use microbial compounds or microbial cells could better be analyzed and solved on a case by case basis.

## Limitations to Compound Use

Despite the potential benefits of microbe derived compounds, there are quite a number of limitations associated with their use.

### Time Consuming

First and foremost, isolation, identification and purification of some compounds is a long and tedious process. This is made worse by the volatile nature of some compounds (Schulz and Dickschat, 2007; Schmidt et al., 2015; Piechulla et al., 2017; Schulz-Bohm et al., 2017), which may necessitate use of sophisticated and perhaps costly isolation technologies. This alone may discourage many researchers from getting involved in work with them.

### Specificity

Some compounds have been reported to address similar stresses across a range of crop species, while others can be quite specific. For instance, carboline, a compound produced by *Elytrigia repens* enhanced resistance to aphids in barley (Bais et al., 2006) but in the absence of barley, the effect of the same compound to aphids was not achieved. Perhaps barley, produces a substance that synergistically works with the compound to enhance tolerance to aphids. Until such questions are answered, through more research, utilization of carboline in aphid control, is likely to be limited to only barley, yet aphids affect a wide range of domesticated plants.

### Requires Proper Control of Concentrations

There is also an issue of concentration. Wrong concentrations, especially high concentrations of many of these compounds, inhibit plant growth, instead of causing growth promotion (Lo Cantore et al., 2015). For instance, Lo Cantore et al. (2015) reported an inhibition in broccoli and lettuce seed germination by DMDS at 2.5 μg, while 0.312 and 0.625 μg of the same compound enhanced growth (Lo Cantore et al., 2015). Some compounds may promote one aspect of plant growth while negatively affecting others. For instance, while 6-pentyl-pyrone, a compound produced by *Trichoderma* spp. suppressed seedling blight, it also led to seedling deformation (El-Hasan and Buchenauer, 2009).

### Antagonism of Useful Soil Microbiome

Worthy of noting are the antagonistic tendencies of some compounds on useful soil microbiome elements and plants, coupled with their ability to enhance growth of plant pathogens (Ryu et al., 2004), which may complicate their use in agriculture. For instance, there have been reports of *Staphylococcus pasteuri* VOCs inhibiting growth of mycorrhizal fungi (Barbieri et al., 2005). Production of hydrogen cyanide (HCN) has been listed as a mechanism through which some biocontrol PGPM (Rijavec and Lapanje, 2016; Nandi et al., 2017) enhance plant growth. However, a study by Blom et al. (2011) indicated that HCN could be connected to the phytotoxicity observed in plants inoculated with PGPM. Groenhagen et al. (2013) reported an increase in antibiotic resistance of *Escherichia coli* exposed to volatile compounds produced by *Burkholderia ambifaria*. This is especially worrying because antibiotic resistance is a characteristic that is undesirable in both animals (including humans) and plant pathology.

### Contradicting Effects the Same Compound

There are also cases of the same compound produced by different microorganisms having opposite effects on plants. For instance, Vaishnav et al. (2016) observed an increase in germination of soybean seeds treated with 50–100 μg of 1-undecene from *Pseudomonas simiae*, while Lo Cantore et al. (2015) and Briard et al. (2016) observed a negative effect on germination of broccoli and lettuce seeds treated with the same VOC, produced by *Pseudomonas aeruginosa*. This leaves one guessing whether the opposite effects are related to the host plants or by the PGPM species. This calls for more research, to have such knowledge gaps filled. For instance, an experiment involving 1-undecene from both species, applied on the same crop species would help solve the puzzle. In the end, it becomes a case by case situation, with farmer preference and a wide range of other factors coming into play.

### Insufficient Knowledge

There is insufficient knowledge regarding how plants perceive some of these compounds (Liu et al., 2012, 2020; Shrestha et al., 2020), which limits their utilization as plant growth stimulants. For instance, in the case of AHLs, plant responses are thought to be are very specific and dependent on the length of the acyl moiety group (Shrestha et al., 2020). While some compounds, which positively affect plant growth may be produced in the natural habitants, sometimes, knowledge of the factors that influence their production remains limited (Blom et al., 2011; van Agtmaal et al., 2015), which makes their production under artificial conditions difficult.

## Way Forward and Conclusion

New microbe derived compounds are being discovered due to ongoing research activities. At this time, quite a number of microbe derived compounds are being utilized in agricultural production, though, the technology has some limitations. Without doubt, a lot more compounds are yet to be discovered given that research in this area is getting more intense (Ledger et al., 2016; Piechulla et al., 2017; Lyu et al., 2020). Given their ability to enhance plant growth it seems clear that microbe derived compounds can play a vital role in sustainable agriculture. Compounds may also work to narrow range of inconsistencies observed following the use of PGPM cells. However, for compound based technology to be more effective, it is necessary that more studies be done, specifically, regarding how they are received and perceived by target organisms, factors, and conditions that influence production of plant growth promoting compounds, and the effects of soil dynamics on the effectiveness of isolated compounds.

## Author Contributions

JN gathered reading material and wrote the review manuscript. DS provided guidance in scientific knowledge and correction of grammatical errors. Both authors contributed to the article and approved the submitted version.

## Conflict of Interest

The authors declare that the research was conducted in the absence of any commercial or financial relationships that could be construed as a potential conflict of interest.
